# Association between dietary sugar intake and depression in US adults: a cross-sectional study using data from the National Health and Nutrition Examination Survey 2011–2018

**DOI:** 10.1186/s12888-024-05531-7

**Published:** 2024-02-08

**Authors:** Lu Zhang, Haiyang Sun, Zihui Liu, Jiguo Yang, Yuanxiang Liu

**Affiliations:** 1 The First Clinical College, Shandong University of Traditional Chinese Medicine, Jinan, China; 2 The Second Clinical Medical College, Guangzhou University of Chinese Medicine, Guangzhou, China; 3 Experimental Center, Shandong University of Traditional Chinese Medicine, Jinan, China; 4 College of Acupuncture and Massage, Shandong University of Traditional Chinese Medicine, Jinan, China; 5 Affiliated Hospital of Shandong University of Traditional Chinese Medicine, Jinan, China

**Keywords:** Dietary sugar intake, Depression, NHANES, Adult, Cross-sectional study

## Abstract

**Background:**

Studies examining whether diet sugar intake increases the risk of depression have produced inconsistent results. Therefore, we investigated this relationship, using the US’ National Health and Nutrition Examination Survey (NHANES) database.

**Methods:**

This cross-sectional study included 18,439 adults (aged ≥ 20 years) from NHANES (2011–2018). Depressive symptoms were assessed using the nine-item version of the Patient Health Questionnaire (PHQ-9). Covariates, including age, sex, race/ethnicity, poverty-income ratio, education, marital status, hypertension, diabetes mellitus, cardiovascular disease, alcohol intake, smoking status, physical activity, and dietary energy intake, were adjusted in multivariate logistic regression models. Subgroup and threshold saturation effect analyses were performed.

**Results:**

After adjusting for potential confounders, we found that a 100 g/day increase in dietary sugar intake correlated with a 28% higher prevalence of depression (odds ratio = 1.28, 95% confidence interval = 1.17–1.40, *P* < 0.001).

**Conclusion:**

Dietary sugar intake is positively associated with depression in US adults.

**Supplementary Information:**

The online version contains supplementary material available at 10.1186/s12888-024-05531-7.

## Background

Depression is a clinically common emotional state characterized by persistently feeling down, losing interest in daily activities, insomnia, and in severe cases, suicidal tendencies [1]. Depression is a significant public health concern. Data from the World Health Organization (WHO) revealed that it affects approximately 4.4% of the global population [2] and projected that depression would be a leading contributor to the global burden of disease by 2030 [3]. Depression not only causes health problems for the patients themselves but also places a heavy economic burden on the family and society. Evidence has demonstrated that early screening and timely intervention can help reduce its severity [4].

The underlying mechanisms are not fully understood. Previous studies have linked dietary factors such as caffeine, fish, and vegetable intake to the risk of depression [5]. However, only a few studies have examined the relationship between dietary sugar intake and depressive symptoms.

Dietary sugars come from a wide range of sources, including those naturally occurring in fruit juices and honey, and those artificially added to drinks or foods [6]. A meta-analysis review of prospective studies showed that associations between dietary factors and depression have been extensively evaluated. A meta-analysis of observational studies found an association between sugar-sweetened beverage consumption and a slightly increased risk of depression. The review also mentions possible biological mechanisms, such as the possibility that sugar intake may affect neurotransmitter production and function, with implications for mood and mental health [7]. In a study that looked at a multi-ethnic sample of people living in Amsterdam, researchers found that the association between a diet high in sugar and saturated fatty acids and depressive symptoms was consistent across ethnic groups. By contrast, no significant association was found between consumption of either the high-sugar dietary pattern or the high-sugar one and depressive symptoms or moods [8]. A prospective study confirmed the adverse effects of sugar intake from sweet foods/beverages on long-term mental health and suggested that lower sugar intake may be associated with better mental health [9]. A multinational study on adults found that patterns of eating processed and rich in sugar foods were associated with a higher risk of depressive symptoms across participating countries [10]. Sugar is a vital source of calories, and excessive intake is associated with an increased risk of obesity, type 2 diabetes, and hypertension (HTN) [11]. It also inhibits the function of the hypothalamic–pituitary–adrenal (HPA) axis—a vital component of the neuroendocrine system—leading to stress and metabolic disorders, such as obesity and diabetes, which can induce oxidative stress and inflammation [12]; HPA axis abnormality and inflammation are important mechanisms underlying depression. Additionally, sugar intake may alter the gut microbiota, which is involved in central nervous system activities such as depression, anxiety, and stress response [13].

Further exploration of the role of dietary sugar intake may help treat depression and its associated complications. This study employed data from the National Health and Nutrition Examination Survey (NHANES) database to explore the association between dietary sugar intake and depression in adults.

## Methods

### Study design and participants

Data for this study were obtained from the NHANES database, a major program conducted by the Centers for Disease Control (CDC) and Prevention to assess the health and nutritional status of individuals in the US [14]. The NHANES contains demographic, dietary, examination, laboratory, and questionnaire data. All study participants provided informed consent and the study protocol was approved by the Ethics Review Board of the National Center for Health Statistics (NCHS). Information can be found on the NHANES website (https://www.cdc.gov/nchs/nhanes/participant.htm).

Participants in our study were screened according to the following inclusion criteria: 1) age 20 years or above, and 2) sugar intake, which was assessed based on a 24-h recall. The exclusion criteria were: 1) incomplete Patient Health Questionnaire-9 (PHQ-9), and 2) no data on dietary sugar intake.

### Assessment of depression

The PHQ-9 is considered an accurate and reliable tool to screen for depression. It contains nine items designed to capture the frequency of depressive symptoms, including appetite problems, fatigue, sleep difficulties, psychomotor retardation or agitation, concentration problems, lack of interest, low mood, feelings of worthlessness, and suicidal thoughts. Each question is scored from ‘0’ (not at all) to ‘3’ (nearly every day), with a total possible score of 0–27; a score ≥ 10 is considered clinically relevant depression (CRD) [15].

### Assessment of dietary sugar intake

Dietary information was collected through interviews. Dietary intake data were used to estimate the types and amounts of foods and beverages (including all types of water) consumed during the 24 h before the interview (midnight to midnight). All NHANES participants were eligible for two 24-h dietary recall interviews. The first meal recall interview was conducted at a mobile screening center (MEC), and the second interview was conducted via telephone 3–10 days later. Interview data files were sent electronically from the field and imported into Survey Net, a computer-assisted food coding and data management system developed by the US Department of Agriculture (USDA) to calculate nutrient intakes. After the intake data were coded, various types of reviews and quality assurance procedures were conducted by the Food Survey Research Group (FSRG) scientists to ensure data quality. The study obtained the daily sugar intake based on the sum of the sugar content of all foods consumed in a single 24-h dietary review. Detailed information regarding the sugar intake assessment can be found at: https://wwwn.cdc.gov/Nchs/Nhanes/2013-2014/DR2TOT_H.htm#DR2TSUGR.

This study extracted the mean sugar intake between the first and second dietary recall as the participants’ dietary sugar intake. For participants who only attended the 24-h dietary recall, sugar intake was defined as the day's sugar intake.

### Assessment of covariates 

The covariates in this study—body mass index (BMI) and energy intake—were used as continuous variables. BMI was measured as weight (kg) divided by height (m) squared. Energy intake was determined by calculating the mean energy intake from the first and second dietary surveys. Categorical variables included age (20–44 years, 45–59 years, ≥ 60 years), sex (male or female), and race/ethnicity (non-Hispanic white, non-Hispanic black, Mexican American, other Hispanic, or other race/multiple races). Education level was categorized as high school not completed, high school completed, high school graduate, or college or associate degrees. Marital status was defined as married/living with a partner or widowed/divorced/separated/never married. Physical activity was self-reported by the participants as lacking, moderate, or vigorous. HTN (defined as systolic blood pressure ≥ 140 mmHg or diastolic blood pressure ≥ 90 mmHg) was determined using three blood pressure measurements at different times, an existing diagnosis, or evidence of an existing antihypertensive medication regimen. Participants were deemed to have diabetes mellitus (DM) if they used glucose-lowering therapies or anti-diabetic medications or had an HbA1c concentration of ≥ 6.5%, an oral glucose tolerance test (OGTT) resulting in ≥ 11.1 mmol/L, fasting plasma glucose ≥ 7.0 mmol/L, or random blood glucose ≥ 11.1 mmol/L. Cardiovascular disease (CVD) was deemed present for participants who experienced or had experienced coronary heart disease, congestive heart failure, heart attack, stroke, or angina. According to smoking habits, participants were categorized as non-smokers (smoked < 100 cigarettes over the lifetime), former smoker (not currently smoking but have consumed ≥ 100 cigarettes previously), and current smoker (smoking ≥ 100 cigarettes in life and smoke every day or some days). According to drinking habits, they were categorized as non-drinkers (drank < 12 drinks over the lifetime), former drinker (drank ≥ 12 drinks a year and has not drunk in the past year, or has not drunk in the past year but has drunk ≥ 12 drinks over the lifetime), and current drinker (has been drinking ≥ 12 drinks in the past year or smoke every day or some days). The poverty-income ratio (PIR) was defined as the ratio of family income to poverty threshold (< 1 indicating an income below the poverty threshold and ≥ 1 indicating an income above the poverty threshold, with the latter category further classified into two groups: 1.00 to < 2.00 and ≥ 2.00).

### Statistical analysis

After adjusting for other factors that may influence depression, the main focus was whether dietary sugar intake was associated with depression. Continuous variables were expressed as mean ± standard deviation, and categorical variables as percentages. The weighted χ2 test was used to compare categorical variables between groups, a one-way analysis of variance to compare normally distributed variables between groups, and the Kruskal–Wallis H test was employed to compare variables with a skewed distribution between groups. A multivariate logistic regression analysis was used to evaluate the independent association between dietary sugar intake and depression. The participants were categorized into four groups based on dietary sugar intake: < 57.47g/d, 57.47 to < 93.42g/d, 93.42 to < 141.76g/d, and ≥ 141.76g/d. The study utilized three levels of adjustment: Model 1 was adjusted for age, sex, and race/ethnicity; Model 2 was adjusted for the variables in Model 1 plus educational level, marital status, and PIR; and Model 3 was adjusted for the variables in Model 2 plus HTN, DM, CVD, drinking status, smoking status, physical activity level, and energy intake. The imputation of missing data was used to impute the missing R package. This random-forest-based technique is computationally highly efficient for high-dimensional data on categorical and continuous predictors [16].

All analyses were performed using the R software (The R Foundation, Vienna, Austria) and Empower (X&Y Solutions, Boston, MA, USA). Statistical significance was defined as a two-sided *P*-value of < 0.05.

## Results

### Participant characteristics

**Fig. 1:**
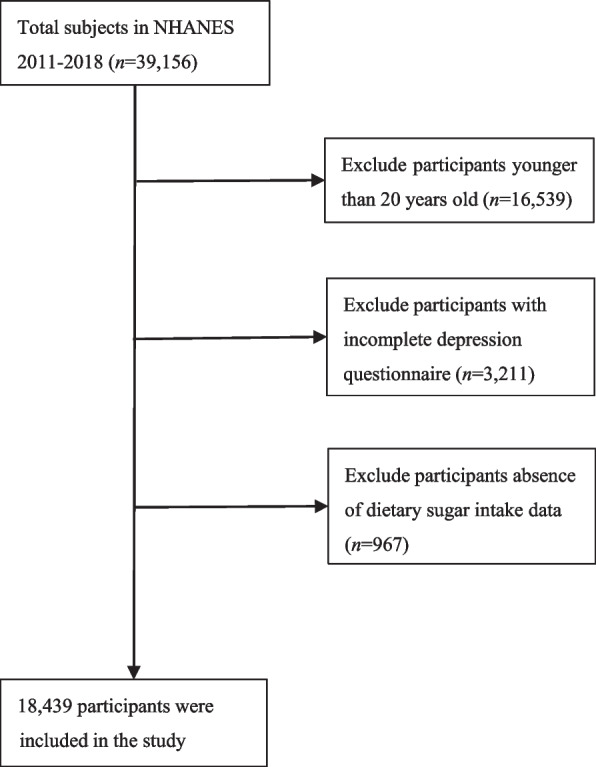
Flowchart for inclusion of study participants

This study included 18,439 participants (Fig. 1).

Table 1 presents the quartiles of participants according to their dietary sugar intake. There were statistically significant differences in age, sex, educational level, race/ethnicity, marital status, PIR, smoking status, alcohol status, HTN, DM, CVD, energy intake, and physical activity among the different dietary sugar intake groups (*P* < 0.05). No significant differences occurred in BMI (*P* > 0.05). Covariates with *P* < 0.05 in univariate analysis were included for further analysis.

**Table 1:** Characteristics of the study population (*N* = 18,439)

Characteristic	Overall	Sugar intake quartiles, g/d				*P*—value
		**Group 1**	**Group 2**	**Group 3**	**Group 4**	
		**(< 57.47 g/d)**	**(≥ 57.47 to < 93.42 g/d)**	**(≥ 93.42 to < 141.76 g/d)**	**(≥ 141.76 g/d)**	
Sample size, n (%)	18,439 (100)	4610 (25.00)	4607 (24.99)	4612 (25.01)	4610 (25.00)	
Age, y, n (%)						< 0.001
20 to < 45	7662 (41.55)	1666 (36.14)	1762 (38.25)	1910 (41.41)	2324 (50.41)	
45 to < 60	4528 (24.56)	1120 (24.30)	1092 (23.70)	1160 (25.15)	1156 (25.08)	
≥ 60	6249 (33.89)	1824 (39.57)	1753 (38.05)	1542 (33.43)	1130 (24.51)	
Sex, n (%)						< 0.001
Male	9073 (49.21)	1991 (43.19)	1963 (42.61)	2291 (49.67)	2828 (61.34)	
Female	9366 (50.79)	2619 (56.81)	2644 (57.39)	2321 (50.33)	1782 (38.66)	
Educational level, n (%)						< 0.001
< High school	3804 (20.64)	1109 (24.06)	908 (19.73)	877 (19.03)	910 (19.74)	
Completed high school	4159 (22.57)	976 (21.18)	1008 (21.90)	1044 (22.65)	1131 (24.53)	
> High school	10,468 (56.80)	2524 (54.76)	2687 (58.37)	2688 (58.32)	2569 (55.73)	
Race/ethnicity, n (%)						< 0.001
Non-Hispanic White	7151 (38.78)	1674 (36.31)	1723 (37.40)	1804 (39.12)	1950 (42.30)	
Non-Hispanic Black	4196 (22.76)	995 (21.58)	980 (21.27)	1066 (23.11)	1155 (25.05)	
Mexican American	2475 (13.42)	573 (12.43)	660 (14.33)	651 (14.12)	591 (12.82)	
Other Hispanic	1886 (10.23)	472 (10.24)	491 (10.66)	482 (10.45)	441 (9.57)	
Other Races	2731 (14.81)	896 (19.44)	753 (16.34)	609 (13.20)	473 (10.26)	
Marital status, n (%)						0.040
Married/living with partner	10,804 (58.62)	2695 (58.51)	2746 (59.63)	2737 (59.38)	2626 (56.96)	
Widowed/ divorced/ separated/ never married	7626 (41.38)	1911 (41.49)	1859 (40.37)	1872 (40.62)	1984 (43.04)	
PIR, n (%)						< 0.001
< 1.00	3580 (21.28)	946 (22.72)	826 (19.77)	783 (18.46)	1025 (24.18)	
1.00 to < 2.00	4511 (26.82)	1074 (25.80)	1094 (26.19)	1139 (26.86)	1204 (28.40)	
≥ 2.00	8729 (51.90)	2143 (51.48)	2257 (54.03)	2319 (54.68)	2010 (47.42)	
BMI, kg/m^2^, mean (SD)	29.47 (7.15)	29.55 (7.21)	29.32 (6.91)	29.56 (7.25)	29.47 (7.21)	0.520
Smoking status, n (%)						< 0.001
Never smoking	10,431 (56.60)	2621 (56.89)	2730 (59.30)	2712 (58.83)	2368 (51.40)	
Former smoker	4402 (23.89)	1148 (24.92)	1154 (25.07)	1113 (24.14)	987 (21.42)	
Current smoker	3595 (19.51)	838 (18.19)	720 (15.64)	785 (17.03)	1252 (27.18)	
Alcohol status, n (%)						< 0.001
Never drinking	2527 (14.47)	741 (17.04)	683 (15.65)	602 (13.75)	501 (11.45)	
Former drinker	2371 (13.58)	545 (12.53)	608 (13.94)	575 (13.13)	643 (14.70)	
Current drinker	12,566 (71.95)	3062 (70.42)	3072 (70.41)	3201 (73.12)	3231 (73.85)	
HTN, n (%)	8087 (43.81)	2249 (48.79)	2052 (44.54)	1981 (42.95)	1796 (38.96)	< 0.001
DM, n (%)	3625 (19.88)	1207 (26.41)	1019 (22.31)	788 (17.30)	611 (13.45)	< 0.001
CVD, n (%)	2003 (10.86)	613 (13.30)	489 (10.61)	499 (10.82)	402 (8.72)	< 0.001
Energy intake, kcal/day, mean (SD)	2115.88 (1003.95)	1455.51 (683.47)	1801.53 (661.85)	2199.02 (734.81)	3007.22 (1188.26)	< 0.001
Physical activity, n (%)						< 0.001
Inactive	9339 (50.65)	2422 (52.54)	2293 (49.77)	2270 (49.22)	2354 (51.06)	
Moderate	4745 (25.73)	1215 (26.36)	1223 (26.55)	1203 (26.08)	1104 (23.95)	
Vigorous	1400 (7.59)	323 (7.01)	345 (7.49)	359 (7.78)	373 (8.09)	
Both moderate and vigorous	2955 (16.03)	650 (14.10)	746 (16.19)	780 (16.91)	779 (16.90)	

*Abbreviations*: *SD* Standard deviation, *PIR* Poverty-income ratio—ratio of family income to poverty threshold, *BMI* Body mass index—calculated as weight in kilograms divided by the square of height in meters, *HTN* Hypertension, *DM* Diabetes mellitus, *CVD* Cardiovascular disease

Participants in the lowest dietary sugar intake in quartile (Q1, < 57.47g/d) were generally older, of female gender, highly educated, non-Hispanic white, living with a partner, rather wealthy, smoked less, drank more, had no HTN, DM, or CVD, had a lower energy intake, and were less active. Conversely, participants with the highest dietary sugar intake in Q4 (≥ 141.76g/d) were likely to be younger, of male gender, highly educated, non-Hispanic white, married or cohabitating, had a lower income, consumed more alcohol, never smoked, had no HTN, DM, or CVD, a higher energy intake and did not engage in physical activity.

A comparison of the characteristics between the participants included and excluded from the analysis is presented in Table S1 in the Supplementary Material (except for participants who were excluded because of their age). The missing data are listed in Table S2.

### Association between dietary sugar intake and depression 

**Fig. 2:**
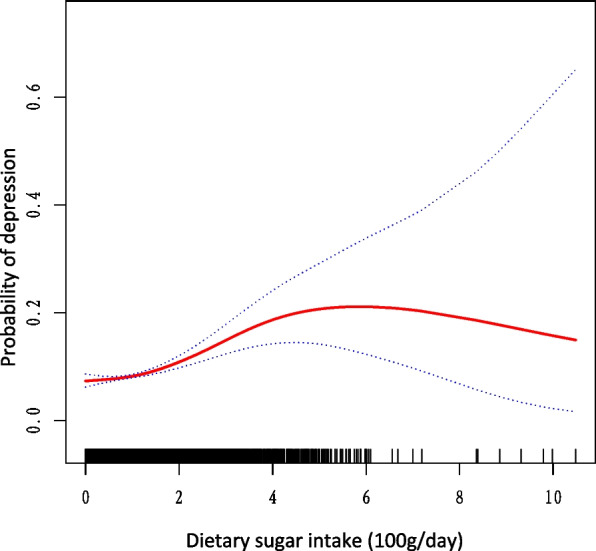
Association between dietary sugar intake and depression in US adults (*n* = 18,439). The black vertical line on the horizontal axis represents the dietary sugar intake distribution, the red line represents the best fit, and the difference between the dashed lines represents the 95% confidence interval. The data were adjusted for age, sex, race/ethnicity, PIR, educational level, marital status, HTN, DM, CVD, drinking status, smoking status, physical activity level, and energy intake

In the comprehensively adjusted model, a linear relationship was observed between dietary sugar intake and depression (Fig. 2).

Table 2 presents the results of the multiple regression analysis. In the crude model, dietary sugar intake was positively correlated with depression (odds ratio [OR] = 1.17, 95% confidence interval [CI]:1.10–1.24, *P* < 0.001). After adjusting for confounders, a significant relationship was observed between dietary sugar intake and depression in Models 1–3. In Model 3, adjustment for all covariates revealed that the incidence of depression increased by 28% for every 100 g/d increase in dietary sugar intake (OR = 1.28, 95% CI = 1.17–1.40, *P* < 0.001).

**Table 2:** Associations of the dietary sugar intake with depression (*N* = 18,439)

	**Crude Model**		**Model 1a**		**Model 2b**		**Model 3c**	
	**OR (95% CI)**	***p*-value**	**OR (95% CI)**	***p*-value**	**OR (95% CI)**	***p*-value**	**OR (95% CI)**	***p*-value**
Per 100 g/day	1.17 (1.10,1.24)	< 0.001	1.23 (1.16,1.30)	< 0.001	1.17 (1.10,1.25)	< 0.001	1.28 (1.17,1.40)	< 0.001
Quartiles								
Q1 (sugar: < 57.47 g/d)	Reference [1]		Reference [1]		Reference [1]		Reference [1]	
Q2 (sugar: ≥ 57.47 to < 93.42 g/d)	0.78 (0.67,0.90)	< 0.001	0.76 (0.66,0.89)	< 0.001	0.82 (0.70,0.96)	0.014	0.87 (0.73,1.03)	0.096
Q3 (sugar: ≥ 93.42 to < 141.76 g/d)	0.83 (0.71,0.95)	0.010	0.84 (0.72,0.97)	0.018	0.91 (0.78,1.06)	0.236	1.01 (0.85,1.20)	0.945
Q4 (≥ 141.76 g/d)	1.11 (0.97,1.27)	0.144	1.21 (1.05,1.39)	0.008	1.21 (1.04,1.41)	0.013	1.33 (1.10,1.61)	0.003
*p* for trend	0.012		< 0.001		< 0.001		< 0.001	

^a^Model 1: Adjusted for age, sex, and race/ethnicity

^b^Model 2: Adjusted for the variables in Model 1 plus poverty-income ratio, educational level, and marital status

^c^Model 3: Adjusted for variables in Model 2 plus hypertension, diabetes mellitus, cardiovascular disease, drinking status, smoking status, physical activity, and energy intake

After adjusting for age, sex, race/ethnicity, PIR, educational level, marital status, HTN, DM, CVD, drinking status, smoking status, physical activity level, and energy intake compared with participants in the first quartile (dietary sugar intake 57.47 g/d), the second group (≥ 57.47 to < 93.42 g/d, OR = 0.87, 95% CI = 0.73–1.03, *P* = 0.096), the third group (≥ 93.42 to < 141.76 g/d, OR = 1.01, 95% CI = 0.85–1.20, *P* = 0.945), and the fourth group (≥ 141.76 g/d, OR = 1.33, 95% CI = 1.10–1.61, *P* = 0.003) had an increased prevalence of depression (*P* for trend was significant in all the models).

**Fig. 3:**
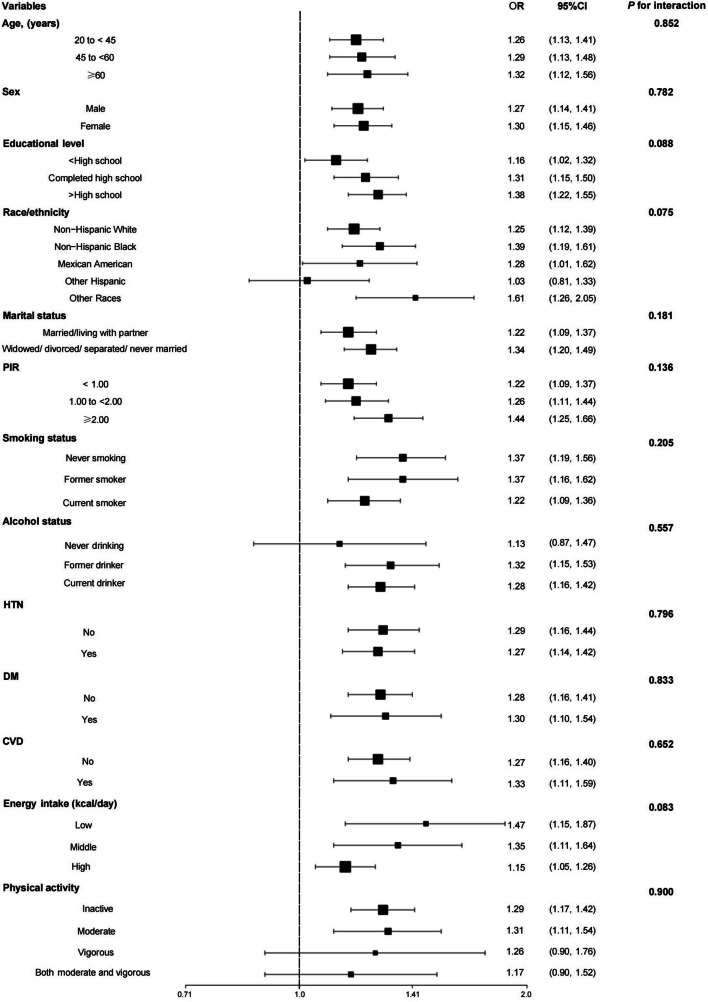
Forest plot of subgroup analysis of the effect of dietary sugar intake on depression (*N* = 18,439) Abbreviations: OR (Odds Ratio); PIR (poverty-income ratio); HTN (hypertension); DM (diabetes mellitus); CVD (cardiovascular disease)

Additionally, we performed subgroup and threshold effect analyses. Subgroup analyses according to age, sex, race/ethnicity, PIR, educational level, marital status, HTN, DM, CVD, drinking status, smoking status, physical activity, and energy intake revealed results similar to those of the main analysis (Fig. 3).

No significant interactions were observed between dietary sugar intake and any of the variables. Table 3 presents the results of the threshold effect, indicating that dietary sugar intake was linearly associated with depression (log-likelihood ratio (LLR) = 0.051).

**Table 3:** Threshold effect analysis for association of dietary sugar intake (100 g/d) with depression

Outcomes	Depression
Model 1, β(95%)	
Linear effort model	1.28 (1.17,1.40)
Model 2, β(95%)	
Infection point (K)	0.74
< K	0.90 (0.63,1.29)
> K	1.33 (1.21,1.47)
LLR	0.051

*Abbreviations*: *LLR* Log-likelihood ratio

## Discussion

This cross-sectional study revealed a positive relationship between dietary sugar intake and depression in American adults. After adjusting for other confounding factors, this relationship was found to be linear, and the risk of depression increased with higher dietary sugar intake.

The details of the mechanisms underlying the relationship between dietary sugar intake and depression need to be explored further, and there may be several possible explanations. Abnormalities in the synthesis and metabolism of monoamine neurotransmitters, which mainly include 5-hydroxytryptamine (5-HT), DA, and norepinephrine (NE), are closely associated with depression [17]. In animal studies, sugar has been found to produce more symptoms of depression than addictive substances; additionally, similarities and overlaps between drug abuse and sugar have been identified [18]. This may be related to dopamine (DA) receptors [19]. It has been found that ginsenosides can significantly increase the levels of 5-HT, NE, DA and metabolite 5-HIAA in the brain of chronic unpredictable mild stimulation (CUMS) model rats, and improve their depression-like behavior by regulating monoamine neurotransmitters [20].

Harrell et al. pointed out that fructose intake can stimulate the HPA axis [21], a vital part of the neuroendocrine system. Hyperfunction of the HPA axis leads to excessive cortisol release and damages feedback inhibition mediated by the glucocorticoid receptor (GR), resulting in the occurrence and development of depression [22].

Experiments have demonstrated that a high-sugar diet can reduce the growth factor and brain-derived neurotrophic factor (BDNF) [23].

Additionally, excessive dietary sugar intake can lead to metabolic disorders and increase the levels of inflammatory mediators and pro-inflammatory cytokines in various tissues [24]. The higher the level of inflammation, the greater the risk of depression and resistance to treatment [25, 26]. Bernier et al. have shown that patients with major depressive disorder (MDD) exhibit higher C-reactive protein (CRP) levels and have a high-fat, high-sugar dietary pattern, which may help maintain inflammatory states [27]. Pro-inflammatory cytokines alter the production, metabolism, and transport of neurotransmitters that synergistically affect mood, including dopamine, glutamate, and serum [28]. Basic studies have shown that microglial NLRP3 inflammasome activation mediates diabetes-induced depression-like behavior by triggering neuroinflammation [29].

The intestinal flora regulates intestinal activity and participates in the regulation of depression, anxiety, and stress responses [13]. A high-sugar diet can disrupt the gut microbiota, leading to depression. A basic study showed that mice fed a high-fructose diet (FruD) exhibited neuroinflammation, decreased hippocampal neurogenesis, and blood–brain barrier (BBB) damage, accompanied by reduced intestinal microbiome derived short-chain fatty acids (SCFA), and that chronic stress exacerbated these pathological changes, promoting the development of depression-like behavior in FruD mice [30]. It can also exaggerate insulin responses, induce hypoglycemia, or increase insulin resistance [31].

Basic research suggests that depression-like behavior caused by type 2 diabetes can be ameliorated by reducing insulin resistance, inflammation, and improving the HPA axis dysfunction [32]. To determine the causal relationship between insulin resistance and the risk of developing depression, in a Dutch cohort study that predicted the incidence of major depression using three measures of insulin resistance, the development of prediabetes between enrollment and the 2-year study visit was positively associated with the occurrence of major depression and positively predicted the occurrence of major depression over the 9-year follow-up period. These findings may have practical value in assessing the risk of developing major depressive disorder in patients with insulin resistance or metabolic pathology [33].

However, it is important to note that this is just an example of some of the existing research and potential mechanisms, and more research is still needed to further confirm and explore the relationship between a high-sugar diet and depression, which is a complex, multifactorial disease; eating habits are only one factor, not the only cause. Therefore, when assessing and exploring risk factors for depression, we should consider a comprehensive set of factors.

Our study revealed increased odds of depression with increased dietary sugar intake in adults, demonstrating that controlling the latter may be beneficial in preventing the former. The findings offer new clues about the potential impact of diet on depression. Emphasis can thus be placed on reducing dietary sugar intake, helping the population to become aware of the link between diet and mental health. Based on our results, health institutions and government agencies can carry out nutritional education and publicity, formulate relevant policies, and provide guidance for the public to live a healthy lifestyle. These can help promote cognitive and behavioral changes in the general public that can improve eating habits, reduce dietary sugar intake, and enhance the overall mental health, thus preventing depression. However, it is important to note that these applications require further research to support this conclusion. Diet needs to be combined with mental health interventions to yield better results.

This study has several limitations. First, a cross-sectional design can only provide data at a point in time and cannot establish causality. When studying the relationship between dietary sugar intake and depression, it is not possible to determine whether the former causes the latter or vice versa. Second, while PHQ-9 is a validated screening tool for assessing the frequency of depressive symptoms, it is not suitable for diagnosing clinical depression. Third, cross-sectional designs make it difficult to control for potentially confounding variables, such as socioeconomic status, genetic factors, and other lifestyle factors. These variables may have an impact on the relationship between dietary sugar intake and depression. Fourth, the differences in demographics and population characteristics in the US may limit the generalizability of the study’s findings to other countries or regions. Fifth, a cross-sectional study cannot determine the temporal order between variables. It is not possible to determine whether depression causes the change in food choice or whether the change in food choice causes the onset of depressive symptoms. Sixth, the study did not provide dietary information on sugar substitutes, such as aspartame. In the future, we will try to conduct further exploration and research about the relationship between the increasing use of sugar substitutes in diet therapy and depression. Further research is needed to confirm the observations in a cross-sectional study, and at a later stage longitudinal study designs or other more effective research methods can be used to further explore the relationship between dietary sugar intake and depression.

## Conclusions

Our research reveals that a higher dietary sugar intake in American adults is positively related to a higher prevalence of depression. Further studies are required to explore the underlying mechanisms and the potential benefits of controlling dietary sugar intake in patients with depression.

### Supplementary Information

**Additional file 1: Table S1. **Characteristics of the included and excluded population. **Table S2. **Missing covariates of study participants (*n* = 18,439).

## Data Availability

No datasets were generated or analysed during the current study.

## Abbreviations

WHO: World Health Organization
HTN: Hypertension
HPA: Hypothalamic-pituitary-adrenal
NHANES: National Health and Nutrition Examination Survey
CDC: Centers for Disease Control
NCHS: National Center for Health Statistics
PHQ-9: Patient Health Questionnaire-9
MEC: Mobile screening center
USDA: US Department of Agriculture
FSRG: Food Survey Research Group
BMI: Body mass index
DM: Diabetes mellitus
OGTT: Oral glucose tolerance test
CVD: Cardiovascular disease
PIR: Poverty-income ratio
OR: Odds ratio
LLR: Log-likelihood ratio
BDNF: Brain-derived neurotrophic factor
SSB: Sugar-sweetened beverage
MDD: Major depressive disorder
CRP: C-reactive protein
