# Supplementary material for: Association between dietary sugar intake and depression in US adults: a cross-sectional study using data from the National Health and Nutrition Examination Survey 2011–2018
Source: BMC Psychiatry. 2024 Feb 8;24:110. doi: 10.1186/s12888-024-05531-7 (PMC10851576; doi:10.1186/s12888-024-05531-7)
Supplement: Supplementary file 1 — Additional file 1: Table S1. Characteristics of the included and excluded population. Table S2. Missing covariates of study participants (n = 18,439). [file 12888_2024_5531_MOESM1_ESM.docx]

**Table S1.** Characteristics of the included and excluded population.

| Characteristic | Excluded | Included | *p*-value |
| --- | --- | --- | --- |
|  |  |  |  |
|  |  |  |  |
| Sample size | 18439 | 5145 |  |
| Age, y, n (%) |  |  | 0.003 |
| 20 to < 45 | 7662 (41.55) | 2115 (41.11) |  |
| 45 to <60 | 4528 (24.56) | 1171 (22.76) |  |
| ≥60 | 6249 (33.89) | 1859 (36.13) |  |
| Sex, n (%) |  |  | <0.001 |
| Male | 9073 (49.21) | 2316 (45.01) |  |
| Female | 9366 (50.79) | 2829 (54.99) |  |
| Educational level, n (%) |  |  | <0.001 |
| <High school | 3804 (20.64) | 1565 (30.56) |  |
| Completed high school | 4159 (22.57) | 1072 (20.93) |  |
| >High school | 10468 (56.80) | 2484 (48.51) |  |
| Race/ethnicity, n (%) |  |  | <0.001 |
| Non-Hispanic White | 7151 (38.78) | 1381 (26.84) |  |
| Non-Hispanic Black | 4196 (22.76) | 1141 (22.18) |  |
| Mexican American | 2475 (13.42) | 712 (13.84) |  |
| Other Hispanic | 1886 (10.23) | 592 (11.51) |  |
| Other Races | 2731 (14.81) | 1319 (25.64) |  |
| Marital status, n (%) |  |  | 0.118 |
| Married/living with partner | 10804 (58.62) | 2946 (57.40) |  |
| Widowed/ divorced/ separated/ never married | 7626 (41.38) | 2186 (42.60) |  |
| PIR, n (%) |  |  | <0.001 |
| < 1.00 | 3580 (21.28) | 1150 (27.32) |  |
| 1.00 to <2.00 | 4511 (26.82) | 1182 (28.08) |  |
| ≥2.00 | 8729 (51.90) | 1878 (44.61) |  |
| Smoking status, n (%) |  |  | <0.001 |
| Never smoking | 10431 (56.60) | 3105 (60.47) |  |
| Former smoker | 4402 (23.89) | 1051 (20.47) |  |
| Current smoker | 3595 (19.51) | 979 (19.07) |  |
| Alcohol status, n (%) |  |  | <0.001 |
| Never drinking | 2527 (14.47) | 8 (0.40%) |  |
| Former drinker | 2371 (13.58) | 288 (14.34) |  |
| Current drinker | 12566 (71.95) | 1712 (85.26) |  |
| HTN, n (%) | 8087 (43.81) | 2118 (41.17) | <0.001 |
| DM, n (%) | 3625 (19.88) | 1011 (19.85) | 0.965 |
| CVD, n (%) | 2003 (10.86) | 630 (12.25) | 0.005 |
| Energy intake, kcal/day, mean (SD) | 2115.88 (1003.95) | 2043.06 (1028.48) | 0.016 |
| Physical activity, n (%) |  |  | <0.001 |
| Inactive | 9339 (50.65) | 2957 (57.48) |  |
| Moderate | 4745 (25.73) | 1180 (22.94) |  |
| Vigorous | 1400 (7.59) | 348 (6.77) |  |
| Both moderate and vigorous | 2955 (16.03) | 659 (12.81) |  |

Abbreviations: SD, standard deviation; PIR, poverty-income ratio (ratio of family income to poverty threshold); HTN, hypertension; DM, diabetes mellitus; CVD, cardiovascular disease.

**Table S2.** Missing covariates of study participants (*n* = 18,439).

| Variable | Number of patients (% missing) |
| --- | --- |
| Age | 0 (0%) |
| Sex | 0 (0%) |
| Race/ethnicity | 0 (0%) |
| Educational level | 8 (0.04%) |
| Marital status | 9 (0.05%) |
| Poverty-income ratio | 1619 (8.78%) |
| Alcohol status | 975 (5.29%) |
| Smoking status | 11 (0.06%) |
| Hypertension | 0 (0%) |
| Diabetes mellitus | 203 (1.10%) |
| Cardiovascular disease | 2 (0.01%) |
| Physical activity | 0 (0%) |
| Energy intake | 0 (0%) |
